# Dietary Xanthan Gum Alters Antibiotic Efficacy against the Murine Gut Microbiota and Attenuates *Clostridioides difficile* Colonization

**DOI:** 10.1128/mSphere.00708-19

**Published:** 2020-01-08

**Authors:** Matthew K. Schnizlein, Kimberly C. Vendrov, Summer J. Edwards, Eric C. Martens, Vincent B. Young

**Affiliations:** a Department of Microbiology and Immunology, University of Michigan, Ann Arbor, Michigan, USA; b Department of Internal Medicine, Division of Infectious Diseases, University of Michigan, Ann Arbor, Michigan, USA; University of Wisconsin—Madison

**Keywords:** *Clostridioides difficile*, dietary fiber, microbial ecology, xanthan gum

## Abstract

Dietary fiber provides a variety of microbiota-mediated benefits ranging from anti-inflammatory metabolites to pathogen colonization resistance. A healthy gut microbiota protects against Clostridioides difficile colonization. Manipulation of these microbes through diet may increase colonization resistance to improve clinical outcomes. The primary objective of this study was to identify how the dietary fiber xanthan gum affects the microbiota and C. difficile colonization. We added 5% xanthan gum to the diet of C57BL/6 mice and examined its effect on the microbiota through 16S rRNA gene amplicon sequencing and short-chain fatty acid analysis. Following either cefoperazone or an antibiotic cocktail administration, we challenged mice with C. difficile and measured colonization by monitoring the CFU. Xanthan gum administration is associated with increases in fiber-degrading taxa and short-chain fatty acid concentrations. However, by maintaining both the diversity and absolute abundance of the microbiota during antibiotic treatment, the protective effects of xanthan gum administration on the microbiota were more prominent than the enrichment of these fiber-degrading taxa. As a result, mice that were on the xanthan gum diet experienced limited to no C. difficile colonization. Xanthan gum administration alters mouse susceptibility to C. difficile colonization by maintaining the microbiota during antibiotic treatment. While antibiotic-xanthan gum interactions are not well understood, xanthan gum has previously been used to bind drugs and alter their pharmacokinetics. Thus, xanthan gum may alter the activity of the oral antibiotics used to make the microbiota susceptible. Future research should further characterize how this and other common dietary fibers interact with drugs.

**IMPORTANCE** A healthy gut bacterial community benefits the host by breaking down dietary nutrients and protecting against pathogens. Clostridioides difficile capitalizes on the absence of this community to cause diarrhea and inflammation. Thus, a major clinical goal is to find ways to increase resistance to C. difficile colonization by either supplementing with bacteria that promote resistance or a diet to enrich for those already present in the gut. In this study, we describe an interaction between xanthan gum, a human dietary additive, and the microbiota resulting in an altered gut environment that is protective against C. difficile colonization.

## Abstract (precis)

A healthy gut bacterial community benefits the host by breaking down dietary nutrients and protecting against pathogens. Clostridioides difficile capitalizes on the absence of this community to cause diarrhea and inflammation. Thus, a major clinical goal is to find ways to increase resistance to C. difficile colonization by either supplementing with bacteria that promote resistance or a diet to enrich for those already present in the gut. In this study, we describe an interaction between xanthan gum, a human dietary additive, and the microbiota resulting in an altered gut environment that is protective against C. difficile colonization.

## INTRODUCTION

The microbiota plays an integral role in gut health by aiding in digestion and regulating colonic physiology (1, 2). Manipulating the microbiota to improve human health by either administering live bacteria (i.e., probiotics) or adding nondigestible, microbiota-accessible ingredients to the host’s diet (i.e., prebiotics) has become a prominent area of biomedical research. While probiotics rely on exogenously added microbes for their effect, diet modification uses indigenous microbes already present in the gut to generate the beneficial effects described above. While the community as a whole may remain intact, diet modification can affect subsets of the community that are better suited to utilize the altered nutrient composition (3). This effect is most prominent in hunter-forager societies where seasonal dietary changes modulate the microbiota (4). In Western diets, a great emphasis has been placed on the types and abundance of host indigestible fiber polysaccharides that are only accessible by the microbiota, such as resistant starch, inulin, or the fibers naturally present in fruits, vegetables, and whole grains.

Dietary fiber promotes microbial short-chain fatty acid (SCFA) production. While SCFA profiles are unique from individual to individual, they provide a variety of benefits, including increased colonic barrier integrity and decreased inflammation (5–10). Depending on the structure of the fiber backbone and side chains, polysaccharides select for unique taxa and, as a result, unique fermentation profiles (11). Several key species may be responsible for degrading the fiber’s carbohydrate structure, the by-products of which go on to be metabolized by a number of additional taxa (12). Butyrate, an SCFA and product of fiber degradation, has been linked to increased gut barrier integrity and decreased inflammation (13–15). Fiber degradation and SCFA production are also associated with clearance of Clostridioides difficile, formerly known as Clostridium difficile, following fecal microbiota transfer (16). Switching mice to a high-fiber diet while colonized with C. difficile increased the SCFA concentrations and also cleared the infection (17). Since C. difficile infection represents a significant health care burden, characterizing how these polysaccharides shape the gut environment and impact C. difficile’s ability to colonize will provide insight into how they might be used to improve patient outcomes.

Some polysaccharides included in food are added to alter texture rather than for nutritional benefit. Xanthan gum, synthesized by the bacterium Xanthomonas campestris, is a common food additive used as a thickener, particularly in gluten-free foods, where industrial production is worth approximately $0.4 billion each year. Xanthan gum structure consists of (1→4)-linked *β*-d-glucose with trisaccharide chains containing two mannose and one glucuronic acid residues linked to every other glucose molecule in the backbone, with possible acetylation on the first branching mannose and 3,6-pyruvylation on the terminal mannose (18). These negatively charged side chains give xanthan gum its viscous, gel-like properties. Although not specifically included in foods for its potential prebiotic activity, bacteria can degrade xanthan gum to increase fecal SCFA concentrations (19, 20). However, little is known about what bacterial taxa are involved in these transformations.

This study investigated the effect of xanthan gum on the bacterial composition of specific-pathogen-free C57BL/6 mice and its effect during an antibiotic model of C. difficile infection. Our goal was to (i) characterize the effects of xanthan gum on the mouse microbiota and (ii) characterize the effects of xanthan gum on C. difficile colonization. Surprisingly, we found that xanthan gum administration alters mouse susceptibility to C. difficile colonization by maintaining the microbiota during antibiotic treatment.

## RESULTS

### Xanthan gum maintains an abundance of microbial taxa during cefoperazone treatment.

**FIG 1:**
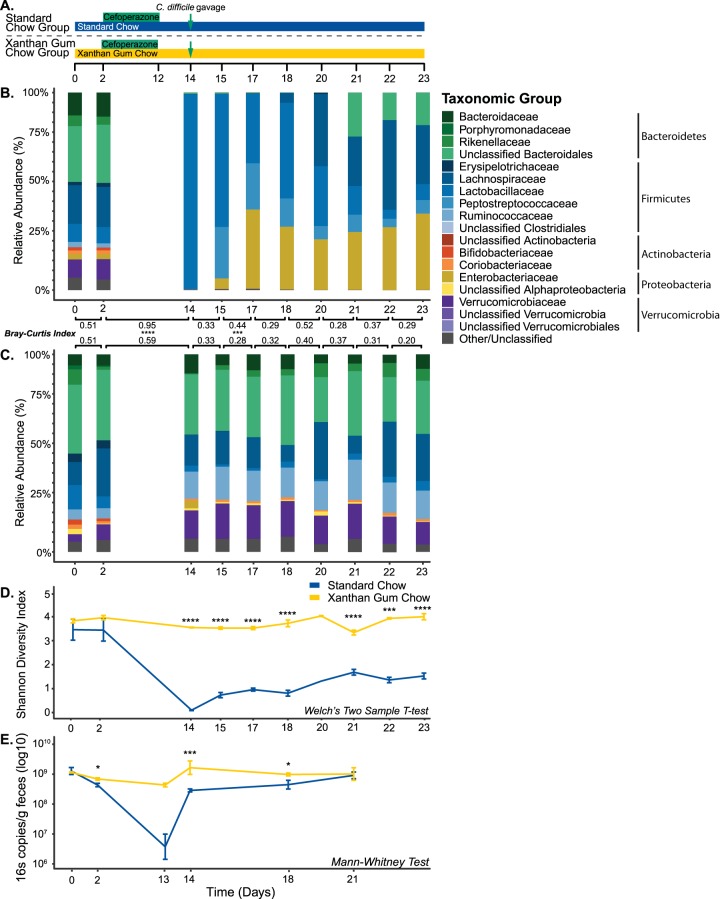
Fecal bacterial diversity and abundance during xanthan gum and cefoperazone administration. (A) Time course of the experimental model for the mice on standard and xanthan gum chows. Mice were challenged with C. difficile on day 14. (B) Microbiota mean relative abundance in mice on standard chow (*n* = 5). (C) Microbiota mean relative abundance in mice on xanthan gum chow (*n* = 6). Bray-Curtis dissimilarity index is shown comparing each time point. (D) Mean Shannon diversity index of the bacterial communities shown in panels B and C (error bars indicate one standard deviation). Statistical testing was performed using Welch’s two-sample *t* test. (E) Bacterial absolute abundance indicated by qPCR using “universal” primers for the 16S rRNA-gene (normalized to grams of feces; error bars indicate one standard deviation). For statistical analysis, a Mann-Whitney test for β-diversity and 16S qPCR, as well as Welch’s two-sample *t* test for Shannon diversity, was performed (*, *P* < 0.05; **, *P* < 0.01; ***, *P* < 0.001; ****, *P* < 0.0001).

Using C57BL/6 mice, we tested the effects of xanthan gum on the microbiota using mouse models designed to study the effects of antibiotic perturbation. Since our initial goal was to study the effects of xanthan gum on C. difficile infection in mice, these models entailed multiple days of antibiotic treatment necessary to make the microbiota susceptible to C. difficile (Fig. 1A; see also Fig. S1A in the supplemental material). Some mice were kept on a standard mouse chow diet; the rest were put on an equivalent diet supplemented with 5% xanthan gum.

10.1128/mSphere.00708-19.1 FIG S1 Fecal (days 0 to 15) and cecal (day 22) bacterial diversity and relative abundance during xanthan gum and antibiotic cocktail administration. (A) Time course of the experimental model for the mice on standard and xanthan gum chows. Mice were challenged with C. difficile on day 14. (B) Microbiota mean relative abundance in mice on standard chow (*n* = 5). (C) Microbiota mean relative abundance in mice on xanthan gum chow (*n* = 7). The Bray-Curtis dissimilarity index is shown comparing each time point. (D) Mean Shannon diversity index of the bacterial communities shown in panels B and C (error bars indicate one standard deviation). Statistical analyses included the Mann-Whitney test for β-diversity, as well as Welch’s two-sample *t* test for Shannon diversity (*, *P* < 0.05; **, *P* < 0.01; ***, *P* < 0.001; ****, *P* < 0.0001). Download FIG S1, EPS file, 0.7 MB. Copyright © 2020 Schnizlein et al. 2020 Schnizlein et al. This content is distributed under the terms of the Creative Commons Attribution 4.0 International license.

In the cefoperazone mouse model, 16S rRNA gene analysis of mouse fecal samples revealed a baseline microbiota dominated by *Bacteroidetes* (∼45%) and *Firmicutes* (∼35%), with the remainder of the community composed of *Actinobacteria*, *Proteobacteria*, and *Verrucomicrobia* (Fig. 1B and C). After cefoperazone treatment of mice on standard chow, *Lactobacillaceae* predominated a fluctuating community, as evidenced by increased mean Bray-Curtis distances between time points. Although we observed higher dissimilarity in the xanthan gum chow group following antibiotics, microbial communities were significantly more similar in the xanthan gum chow group compared to the standard chow group, as measured by Bray-Curtis distances. These data also indicated that by day 23 the microbial community in the standard chow group had not returned to the preantibiotic baseline (mean Bray-Curtis distance from day 2 to day 23, 0.86) compared to the xanthan gum chow group (mean Bray-Curtis distance from day 2 to day 23, 0.52). However, the relative abundance of bacterial taxa remained similar after cefoperazone treatment in mice fed 5% xanthan gum (Fig. 1C). These protective effects are reflected in a significantly higher Shannon diversity and an absolute abundance of fecal bacteria in xanthan gum-fed mice following cefoperazone treatment compared to those on standard chow (Fig. 1D and E). We observed similar antimicrobial activity against an Escherichia coli strain ECOR2 lawn from fecal extracts obtained during antibiotic administration between diet groups and no inhibitory activity in fecal extracts from non-antibiotic-treated control mice (see Fig. S5B in the supplemental material). These data suggest that high concentrations of xanthan gum prevent cefoperazone-mediated alterations of the mouse microbiota. To determine whether xanthan gum had a similar protective effect for other antibiotics, we also used an oral antibiotic cocktail model coupled with intraperitoneal (i.p.) clindamycin, which has also been shown to render mice susceptible to C. difficile colonization (21, 22). However, the microbiota differences between chow groups were less pronounced (Fig. S1B and C). Taken together, our results show that xanthan gum administration maintains both diversity and overall abundance of microbes in the gut during cefoperazone treatment.

Using the linear discriminant analysis effect size method (LEfSe), we identified 35 operational taxonomic units (OTU) that were significantly increased 2 days following the switch from standard to xanthan gum chow (Fig. S3). We also observed a shift in bacterial metabolism marked by significantly higher butyrate and propionate concentrations in mice on xanthan gum chow compared to those on standard chow (Fig. S4). No OTU abundances were identified as being significantly different when comparing the same time points in the standard chow group. After cefoperazone treatment, 4 OTU were increased and 80 OTU were decreased in the xanthan gum group (Fig. S4). In the standard chow group, only 1 OTU (*Lactobacillus*) significantly increased following cefoperazone treatment (Fig. S6). Unsurprisingly, 48 of the 112 OTU that were negatively correlated with cefoperazone treatment in the standard chow group were also negatively correlated in the xanthan gum group.

### Xanthan gum-mediated microbiota protection limits *C. difficile* colonization.

**FIG 2:**
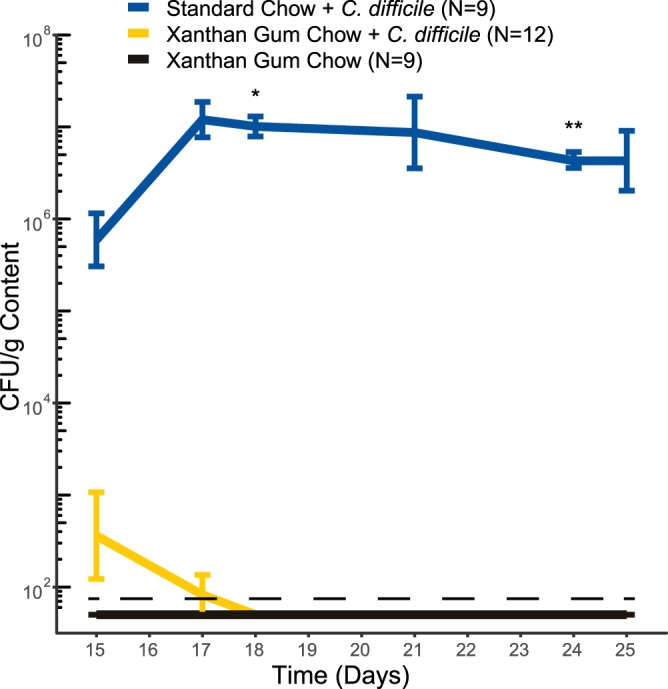
C. difficile colonization in mice on standard and xanthan gum chows. C. difficile CFU in cefoperazone-treated mice were normalized to the fecal mass. The lines indicate the mean CFU levels (error bars indicate one standard deviation). The data shown are from experiments 1 and 2. Statistical testing was performed using Welch’s two-sample *t* test (*, *P* < 0.05; **, *P* < 0.01).

Two days after the mice were removed from cefoperazone, they were challenged with C. difficile strain 630g spores administered by oral gavage. By monitoring feces for CFU (both vegetative cells and spores), we observed approximately 1 × 10^6^ CFU/g feces C. difficile in mice on standard chow 1 day postgavage (day 15), which rose to 1 × 10^7^ for the duration of the experiment (Fig. 2). However, when on xanthan gum, only a small number of CFU was observed 1 day postgavage, but by day 4 (day 19) all mice had cleared C. difficile (Fig. 2). In the antibiotic cocktail model, C. difficile colonized mice on both standard and xanthan gum chow similarly until 7 days postgavage (day 15) when C. difficile colonization levels were significantly lower in the mice on xanthan gum chow (Fig. S6).

## DISCUSSION

The use of dietary polysaccharides for their beneficial health effects, either directly on the host or indirectly through the microbiota, has been widely demonstrated (15, 19). In the context of C. difficile, diet may play a role in pathogen evolution, such as with trehalose, or influence colonization resistance, such as with dietary fiber and zinc (17, 23–25). Dietary alteration may shape the intestinal environment by altering the nutrients available or by modulating the concentrations of compounds toxic to C. difficile, such as secondary bile salts. As a common food additive, xanthan gum’s physicochemical properties are well known (18). However, its effects on the gut microbiota are poorly understood. Although we were not able to test whether xanthan gum enriches for fiber-degrading bacteria to increase colonization resistance, we did observe that xanthan gum interferes with the activity of orally administered antibiotics to protect mice from C. difficile colonization. These protective effects vary by type of antibiotic. While xanthan gum may have enriched for taxa capable of degrading it, these changes were minor compared to the much larger differences observed between diet groups during antibiotic treatment.

As a third-generation cephalosporin, cefoperazone has broad-spectrum efficacy (26, 27). As a result, it is not surprising that, in the standard chow group, it had a significant impact on microbiota community structure. These results agree with previously published work on cefoperazone’s ability to disrupt the murine gut microbiota and cause lasting alterations even 6 weeks after the cessation of treatment (28, 29). As demonstrated here, diet can affect antibiotic efficacy in unexpected ways. Although both bacterial diversity and abundance were maintained in mice on xanthan gum, the similarities in OTU levels identified by LEfSe between the two groups indicates that cefoperazone affected the microbiota in both groups but was attenuated in the xanthan gum chow group. Since we observed similar antimicrobial activity in feces from each diet group, our data suggest that cefoperazone is still active in the feces from these mice. We also demonstrated that xanthan gum chow itself did not have any inhibitory effect directly on C. difficile (data not shown). These data indicate that cefoperazone retained antibiotic activity in the presence of xanthan gum, but its effect on the microbiota *in vivo* was somehow interfered with. This decreased antibiotic activity in the gut of xanthan gum-fed mice allowed the bacterial community to recover faster than in animals on standard chow.

By at least partially protecting the microbiota from the effects of cefoperazone, xanthan gum administration preserved colonization resistance to C. difficile. Colonization resistance comprises a variety of mechanisms, including the metabolism of bile salts and competition for nutrients (30). Microbially modified secondary bile salts inhibit C. difficile outgrowth much more than their primary precursors (31). Microbial metabolism mediates a variety of modifications to primary bile salts, including deconjugation by *Lactobacillus* and *Bifidobacterium* species, as well as 7α-dehydroxylation by *Clostridium* species (32–35). The lack of secondary metabolites produced by these taxa has been correlated with a lack of colonization resistance (31, 36–38). The indigenous microbiota also prevents C. difficile from establishing itself within the colonic environment by limiting the nutrients available for growth (39, 40). A number of taxa, including the *Lachnospiraceae*, have been shown to provide resistance to C. difficile colonization, which may occur through niche competition (41, 42). Despite increased SCFA concentrations immediately following xanthan gum administration, direct alterations of the microbiota by xanthan gum did not appear to affect colonization resistance on the day of C. difficile gavage since SCFA concentrations had returned to baseline levels. By protecting the microbiota during antibiotic treatment, xanthan gum likely maintained these metabolic mechanisms to exclude C. difficile from the gut. This suggested that while the community was altered, enough bacterial taxa remained to exclude C. difficile.

While we did not demonstrate a mechanism for xanthan gum’s effect, its gel-like nature may interrupt the activity of antibiotics by altering their pharmacokinetics. Several large polysaccharides with negatively charged or polar side chains, such as hydroxypropylmethyl cellulose, mannan oligosaccharides, and guar gum, increase the excretion of cholesterol and bile salts in feces by limiting their absorption (43–49). While not previously reported, xanthan gum may also interact with these compounds. Similarities between the chemical structures of these sterol ring-containing compounds and of cefoperazone may result in interactions between xanthan gum and the antibiotic. The greater efficacy of the antibiotic cocktail plus clindamycin model against the microbiota is likely due to varied interactions with the five antibiotics in addition to the effect of the i.p. injection of clindamycin. Although potential alterations to the bile salt pool by xanthan gum may have limited C. difficile germination, we observed more fecal CFU 1 day postgavage (day 15) than what we used to inoculate the mice on day 14, suggesting that any disruptions to enterohepatic circulation did not prevent germination since there was some vegetative cell outgrowth. Furthermore, we have previously observed that few spores (i.e., <100) are sufficient to infect antibiotic-treated mice, suggesting that even if only a limited number of spores germinated, the mice would still become infected (unpublished data). If xanthan gum did alter bile salt concentrations, the resulting changes would be more likely involved in inhibiting vegetative cells instead of preventing germination, since some spores would likely germinate in spite of the bile salt changes.

Polysaccharide-drug interactions are frequently explored as means to delay drug release *in vivo*. When mice consume xanthan gum in their chow, orally administered antibiotics may become trapped inside the gel formed by hydrated xanthan gum. Previous research has shown that xanthan gum would provide time-dependent release that occurs at a lower rate than other large, polar polysaccharides. For example, hydroxypropylmethyl cellulose requires three times the concentration to achieve similar drug binding levels as xanthan gum (50, 51). The binding affinity of xanthan gum is pH-dependent, where a higher pH limits drug release due to the increased integrity of the polymer structure (51). Furthermore, environments with a higher ionic strength, as well as the presence of other polysaccharides, increases xanthan gum’s drug retaining efficiency (52, 53). Thus, the colonic environment would be conducive for high xanthan gum affinity for binding compounds such as cefoperazone due to its relatively higher pH.

In our study, dietary xanthan gum administration protected the microbiota during antibiotic treatment, leading to the exclusion of C. difficile from the gut. Although our study suggests that a common dietary polysaccharide interacts with the effects of antibiotics, there are several limitations that merit future research. Since few individuals will consume xanthan gum at the concentrations we used, titering in lower doses of xanthan gum to get closer to physiological levels would elucidate the effects of xanthan gum in a normal human diet. Future research should also characterize how polar polysaccharides such as xanthan gum interact with compounds in the gut. This would be important for understanding drug pharmacokinetics, as well as the impact of xanthan gum on bile salts and enterohepatic circulation. Further work characterizing this common food additive would provide a greater understanding not only of how it is degraded in the gut it but also the potential positive effects of its fermentative by-products.

## MATERIALS AND METHODS

### Ethics statement.

The University Committee on Use and Care of Animals of the University of Michigan, Ann Arbor, approved all animal protocols used in the present study (PRO00008114). These guidelines comply with those set by the Public Health Service policy on Humane Care and Use of Laboratory Animals.

### Animals and housing.

We obtained 5- to 8-week-old male and female mice from an established breeding colony at the University of Michigan. These mice were originally sourced from Jackson Laboratory. We housed mice in specific-pathogen-free and biohazard AALAC-accredited facilities maintained with 12-h light/dark cycles at an ambient temperature of 22°C ± 2°C. All bedding and water were autoclaved. Mice received gamma-irradiated food (LabSupply 5L0D PicoLab Rodent Diet, a gamma-irradiated version of LabSupply 5001 Rodent LabDiet) or an equivalent diet with 5% xanthan gum added (95% LabSupply 5001 Rodent LabDiet, 5% xanthan gum [Sigma]; gamma-irradiated by manufacturer). We housed mice in groups of two to five animals per cage, with multiple cages per treatment group.

All cage changes, infection procedures, and sample collections were conducted in a biological safety cabinet (BSC) using appropriate sterile personal protective equipment between cage contacts. The BSC was sterilized with Perisept (Triple S, Billerica, MA) between treatment groups. Gloves were thoroughly sprayed with Perisept between each cage and completely changed between groups. A description of the metadata for the mouse experiments, including the cage numbers and treatment groups, can be found in Table S1 in the supplemental material.

10.1128/mSphere.00708-19.7 TABLE S1 Mouse experiment and analysis metadata. Download Table S1, XLSX file, 0.01 MB. Copyright © 2020 Schnizlein et al. 2020 Schnizlein et al. This content is distributed under the terms of the Creative Commons Attribution 4.0 International license.

### Xanthan gum-cefoperazone mouse model.

To investigate the effect of xanthan gum on cefoperazone-treated mice, we switched mice to a diet containing 5% xanthan gum on day zero. Two days later, we gave mice 0.5 mg/ml cefoperazone (MP Biomedicals, Solon, OH) in the drinking water for 10 days, as previously described, to render the mice susceptible to C. difficile colonization (54, 55). We changed the antibiotic-water preparation every 2 days. After 10 days of cefoperazone treatment, we switched mice to Gibco distilled water. We orally gavaged mice with between 10^2^ and 10^4^ C. difficile 630g spores or vehicle control (sterile water) 2 days after removing the mice from antibiotics. Spores were prepared as previously described and then suspended in 200 μl of Gibco distilled water and heat shocked (55). Viable spores were quantified immediately after gavage using taurocholate cycloserine cefoxitin fructose agar (TCCFA) as previously described (55). To monitor infection severity, mice were weighed over the course of the model.

### Xanthan gum-antibiotic cocktail mouse model.

To investigate the effect of xanthan gum on an alternative antibiotic model (antibiotic cocktail with clindamycin), we switched mice to a 5% xanthan gum diet on day 0 and then put on an antibiotic cocktail (0.4 mg/ml kanamycin, 0.035 mg/ml gentamicin, 850 U/ml colistin, 0.215 mg/ml metronidazole, and 0.045 mg/ml vancomycin; Sigma-Aldrich) for 3 days in their drinking water, as previously described (21, 22). On day 5, we removed mice from oral antibiotic administration and returned them to regular drinking water. On day 7, mice were given an i.p. injection of clindamycin hydrochloride (10 mg/kg). One day after the i.p. injection, we orally gavaged mice with between 10^2^ and 10^4^ C. difficile 630g spores and weighed the mice as described above.

### Quantitative culture.

We suspended fresh fecal pellets in sterile, prereduced Gibco phosphate-buffered saline (PBS; Thermo Fisher) using a ratio of 1 part feces to 9 parts Gibco PBS (wt/vol; Thermo Fisher, Waltham, MA). We serially diluted these suspensions, plated them on TCCFA, and then incubated the plates anaerobically at 37°C for 18 to 24 h before counting the colonies.

### Fecal cefoperazone activity assay.

We used fecal supernatant obtained from mice 6 days after the beginning of cefoperazone treatment (day 8). The fecal content was diluted by a factor of 10 in PBS to test its activity on a lawn of Escherichia coli strain ECOR2, which is susceptible to cefoperazone. Next, 10 μl of supernatant was added to a 0.7-cm-diameter autoclaved Whatman filter paper (Sigma-Aldrich) disk and laid in duplicate onto a Luria-Bertani (LB) agar plate (BD Difco, Miller) streaked for lawn growth of E. coli. After incubation of the plates anaerobically at 37°C for 24 h, we measured the zones of inhibition (ZOI) and then confirmed these measurements after another 24 h of anaerobic growth. The ZOIs from samples were compared to those of fecal supernatant from mice not on antibiotics and to PBS controls.

### Lipocalin-2 ELISA.

Fecal supernatants were diluted 100-fold in PBS plus 0.1% Tween 20 (USB Corp., Cleveland, OH) and then tested using the standard protocol for the DuoSet enzyme-linked immunosorbent assay (ELISA) kit for Mouse Lipocalin-2/NGAL (R&D Systems, Minneapolis, MN). Sample concentrations were normalized to g of feces and analyzed in duplicate.

### *E. coli* growth curve with cefoperazone.

We grew E. coli strain ECOR2 overnight in LB broth (Difco LB Broth, Lennox; BD), pelleted the culture, and then resuspended it in fresh LB medium. We back-diluted this bacterial suspension into LB medium or LB medium containing 0.25% xanthan gum. Finally, we added cefoperazone to the growth medium before placing the cultures in a Sunrise microplate reader (Tecan, Switzerland) and monitoring growth for 48 h. Measurements of the optical density at 600 nm were automatically taken every 15 min, with 60 s of shaking immediately prior to measurement.

### 16S rRNA gene qPCR.

We suspended fecal pellets in PBS as described above and centrifuged them at 6,000 rpm for 1 min. Then, 100 to 400 μl of supernatant was removed for metabolite analysis. Using the sedimented fecal content, we performed DNA extractions using a DNeasy PowerSoil kit (Qiagen, Germantown, MD), according to the manufacturer’s protocol. We immediately stored extracted DNA at −20°C until further use. We then performed qPCR on a LightCycler 96 thermocycler (Roche, Basel, Switzerland) using PrimeTime gene expression master mix (IDT, Coralville, IA) and a set of broad-range 16S rRNA gene primers (56). All fecal DNA was amplified in triplicate with E. coli genomic DNA standards in duplicate and negative controls in triplicate. The LightCycler reaction conditions were as follows: 95°C for 3 min, followed by 45 cycles of two-step amplification at 60°C for 60 s and 95°C for 15 s. The quantification cycle (*C_q_*) values for each reaction were determined by using the LightCycler 96 software, and fecal DNA concentrations were determined by comparing *C_q_* values to the standards in each plate and normalizing them to each individual sample’s fecal mass. We used Welch’s two-sample *t* test to determine significance.

### Short-chain fatty acid analysis.

Portions (100 μl) of fecal supernatants were filtered at 4°C using 0.22-μm 96-well filter plates and stored at –80°C until analysis. We transferred the filtrate to 1.5-ml screw cap vials with 100-μl inserts for high-performance liquid chromatography analysis (HPLC) and then randomized them. We quantified acetate, propionate, and butyrate concentrations using a refractive index detector as part of a Shimadzu HPLC system (Shimadzu Scientific Instruments, Columbia, MD) as previously described (8). Briefly, we used a 0.01 N H_2_SO_4_ mobile phase through an Aminex HPX87H column (Bio-Rad Laboratories, Hercules, CA). The sample areas under the curve were compared to volatile fatty acid standards with concentrations of 40, 20, 10, 5, 2.5, 1, 0.5, 0.25, and 0.1 mM. Through blinded curation, we assessed baseline and peak quality and excluded poor-quality data where necessary.

### DNA extraction and Illumina MiSeq sequencing.

The detailed protocol for DNA extraction and Illumina MiSeq sequencing was followed as described in previous publications with modifications (55). Briefly, 200 to 300 μl of 10-fold-diluted fecal pellets were submitted for DNA isolation using a MagAttract PowerMicrobiome DNA isolation kit (Qiagen, Germantown, MD). Samples were randomized into each extraction plate. To amplify the DNA, we used barcoded dual-index primers specific to the V4 region of the 16S rRNA-gene (57). Negative and positive controls were run in each sequencing plate. Libraries were prepared and sequenced using the a 500-cycle MiSeq V2 reagent kit (Illumina, San Diego, CA). Raw FASTQ files, including the appropriate controls, were deposited in the Sequence Read Archive (SRA) database (accession numbers SRX6897486 to SRX6897789).

### Data processing and microbiota analysis.

16S rRNA gene sequencing was performed as previously described using the V4 variable region and analyzed using mothur. Detailed methods, processed read data, and data analysis code are described on GitHub (https://github.com/mschnizlein/xg_microbiota). Briefly, after assembly and quality control, such as filtering and trimming, we aligned contigs to the Silva v.128 16S rRNA database. We removed chimeras using UCHIME and excluded samples with fewer than 5,000 sequences. We binned contigs by 97% percent similarity (OTU) using Opticlust and then used the Silva rRNA sequence database to classify those sequences. Alpha- and beta-diversity metrics were calculated from unfiltered OTU samples. We used LEfSe to identify OTU that significantly associated with changes across diets and antibiotic treatments (58). We performed all statistical analyses in R (v3.5.2).

### Data availability.

Raw FASTQ files are available via the SRA (BioProject ID PRJNA573932; BioSample IDs SAMN12833230 to SAMN12833532). Code and detailed processing information, as well as raw data are available on GitHub (https://github.com/mschnizlein/xg_microbiota).

10.1128/mSphere.00708-19.2 FIG S2 LEfSe analysis of the microbiota of mice before and after the start of xanthan gum administration. (A and B) Bacterial taxa that were increased (A) or decreased (B) after the switch to xanthan gum. The numbers in parentheses indicate the number of OTU that fall under that particular taxonomic classification. Download FIG S2, EPS file, 0.7 MB. Copyright © 2020 Schnizlein et al. 2020 Schnizlein et al. This content is distributed under the terms of the Creative Commons Attribution 4.0 International license.

10.1128/mSphere.00708-19.3 FIG S3 Short-chain fatty acid analysis (acetate, propionate, and butyrate) of mouse fecal content from the cefoperazone model. Time points show before (day 0) and after diet change (day 2), as well as after antibiotic treatment (days 13 and 14). Statistics were performed using Welch’s two-sample *t* test (**, *P* < 0.01). Download FIG S3, EPS file, 0.4 MB. Copyright © 2020 Schnizlein et al. 2020 Schnizlein et al. This content is distributed under the terms of the Creative Commons Attribution 4.0 International license.

10.1128/mSphere.00708-19.4 FIG S4 LEfSe analysis of the microbiota in mice on xanthan gum (A and B) and standard chows (C and D) during cefoperazone treatment. Bacterial taxa that were increased (A and C) or decreased (B and D) following cefoperazone treatment. The numbers in parentheses indicate the number of OTU that fall under that particular taxonomic classification. Download FIG S4, EPS file, 0.8 MB. Copyright © 2020 Schnizlein et al. 2020 Schnizlein et al. This content is distributed under the terms of the Creative Commons Attribution 4.0 International license.

10.1128/mSphere.00708-19.5 FIG S5 Investigating the effect of xanthan gum in the C. difficile mouse model. (A) Escherichia coli strain ECOR2 was grown in LB medium with concentrations of cefoperazone and low levels of xanthan gum. The line represents the average of three technical replicates and two biological replicates. (B) Fecal cefoperazone activity was measured from fecal supernatants at day 8. The diameters of the zones of inhibition are plotted versus the dietary group. Supernatants from cefoperazone-treated mice on standard chow and xanthan gum chow were compared with healthy mouse fecal supernatant not on cefoperazone. Statistical analysis using Welsh’s two-sample *t* test was performed (n.s., not significant). Each dot represents the average of two technical replicates. (C and D) Fecal lipocalin-2 was measured from fecal supernatants from mice in both the cefoperazone (C) and antibiotic cocktail (D) models. Each dot represents the average of two technical replicates. (E and F) Mouse body weight was measured and is presented as a percentage of body weight on the day of C. difficile gavage in the cefoperazone (E) and antibiotic cocktail (F) models. Download FIG S5, EPS file, 0.9 MB. Copyright © 2020 Schnizlein et al. 2020 Schnizlein et al. This content is distributed under the terms of the Creative Commons Attribution 4.0 International license.

10.1128/mSphere.00708-19.6 FIG S6 C. difficile CFU in mice in the antibiotic cocktail model. C. difficile CFU levels in antibiotic cocktail-treated mice were normalized to fecal mass. Lines indicate mean CFU levels (error bars indicate one standard deviation). The data shown are from both experiments 3 and 4. Statistical testing was performed using Welch’s two-sample *t* test (*, *P* < 0.05). Download FIG S6, EPS file, 1.2 MB. Copyright © 2020 Schnizlein et al. 2020 Schnizlein et al. This content is distributed under the terms of the Creative Commons Attribution 4.0 International license.

10.1128/mSphere.00708-19.8 TABLE S2 Mouse sequencing metadata from the cefoperazone model. Download Table S2, CSV file, 0.3 MB. Copyright © 2020 Schnizlein et al. 2020 Schnizlein et al. This content is distributed under the terms of the Creative Commons Attribution 4.0 International license.

10.1128/mSphere.00708-19.9 TABLE S3 Mouse sequencing metadata from the antibiotic cocktail with clindamycin model. Download Table S3, CSV file, 0.3 MB. Copyright © 2020 Schnizlein et al. 2020 Schnizlein et al. This content is distributed under the terms of the Creative Commons Attribution 4.0 International license.

10.1128/mSphere.00708-19.10 TABLE S4 LEfSe metadata from the comparison of bacterial communities before and after the start of xanthan gum administration (xgdiet), as well as cefoperazone treatment in the xanthan gum (xgcef) and standard chow groups (stdcef). Download Table S4, CSV file, 0.03 MB. Copyright © 2020 Schnizlein et al. 2020 Schnizlein et al. This content is distributed under the terms of the Creative Commons Attribution 4.0 International license.

## References

[1] Feng Q, Chen W-D, Wang Y-D 2018 Gut microbiota: an integral moderator in health and disease. Front Microbiol 9:151. doi:10.3389/fmicb.2018.00151. 29515527 PMC5826318

[2] Pan W-H, Sommer F, Falk-Paulsen M, Ulas T, Best P, Fazio A, Kachroo P, Luzius A, Jentzsch M, Rehman A, Müller F, Lengauer T, Walter J, Künzel S, Baines JF, Schreiber S, Franke A, Schultze JL, Bäckhed F, Rosenstiel P 2018 Exposure to the gut microbiota drives distinct methylome and transcriptome changes in intestinal epithelial cells during postnatal development. Genome Med 10:27. doi:10.1186/s13073-018-0534-5. 29653584 PMC5899322

[3] David LA, Materna AC, Friedman J, Campos-Baptista MI, Blackburn MC, Perrotta A, Erdman SE, Alm EJ 2014 Host lifestyle affects human microbiota on daily timescales. Genome Biol 15:R89. doi:10.1186/gb-2014-15-7-r89. 25146375 PMC4405912

[4] Smits SA, Leach J, Sonnenburg ED, Gonzalez CG, Lichtman JS, Reid G, Knight R, Manjurano A, Changalucha J, Elias JE, Dominguez-Bello MG, Sonnenburg JL 2017 Seasonal cycling in the gut microbiome of the Hadza hunter-gatherers of Tanzania. Science 357:802–806. doi:10.1126/science.aan4834. 28839072 PMC5891123

[5] Desai MS, Seekatz AM, Koropatkin NM, Kamada N, Hickey CA, Wolter M, Pudlo NA, Kitamoto S, Terrapon N, Muller A, Young VB, Henrissat B, Wilmes P, Stappenbeck TS, Nuñez G, Martens EC 2016 A dietary fiber-deprived gut microbiota degrades the colonic mucus barrier and enhances pathogen susceptibility. Cell 167:1339–1353.e21. doi:10.1016/j.cell.2016.10.043. 27863247 PMC5131798

[6] Jefferson A, Adolphus K 2019 The effects of intact cereal grain fibers, including wheat bran on the gut microbiota composition of healthy adults: a systematic review. Front Nutr 6:33. doi:10.3389/fnut.2019.00033. 30984765 PMC6449473

[7] Gong L, Cao W, Chi H, Wang J, Zhang H, Liu J, Sun B 2018 Whole cereal grains and potential health effects: involvement of the gut microbiota. Food Res Int 103:84–102. doi:10.1016/j.foodres.2017.10.025. 29389647

[8] Baxter NT, Schmidt AW, Venkataraman A, Kim KS, Waldron C, Schmidt TM, Baxter NT, Schmidt AW, Venkataraman A, Kim KS, Waldron C, Schmidt TM 2019 Dynamics of human gut microbiota and short-chain fatty acids in response to dietary interventions with three fermentable fibers. mBio 10:e02566-18. doi:10.1128/mBio.02566-18. 30696735 PMC6355990

[9] Venkataraman A, Sieber JR, Schmidt AW, Waldron C, Theis KR, Schmidt TM, Tremaroli V, Backhed F, Flint HJ, Duncan SH, Scott KP, Louis P, Hartstra AV, Bouter KE, Backhed F, Nieuwdorp M, Buffie CG, Bucci V, Stein RR, McKenney PT, Ling L, Gobourne A, Donohoe DR, Garge N, 2016 Variable responses of human microbiomes to dietary supplementation with resistant starch. Microbiome 4:33–33. doi:10.1186/s40168-016-0178-x. 27357127 PMC4928258

[10] Koh A, De Vadder F, Kovatcheva-Datchary P, Bäckhed F 2016 From dietary fiber to host physiology: short-chain fatty acids as key bacterial metabolites. Cell 165:1332–1345. doi:10.1016/j.cell.2016.05.041. 27259147

[11] Warren FJ, Fukuma NM, Mikkelsen D, Flanagan BM, Williams BA, Lisle AT, Ó Cuív P, Morrison M, Gidley MJ 2018 Food starch structure impacts gut microbiome composition. mSphere 3:e00086-18. doi:10.1128/mSphere.00086-18. 29769378 PMC5956147

[12] Shortt C, Hasselwander O, Meynier A, Nauta A, Fernández EN, Putz P, Rowland I, Swann J, Türk J, Vermeiren J, Antoine J-M 2018 Systematic review of the effects of the intestinal microbiota on selected nutrients and non-nutrients. Eur J Nutr 57:25–49. doi:10.1007/s00394-017-1546-4. 29086061 PMC5847024

[13] Park CH, Eun CS, Han DS 2018 Intestinal microbiota, chronic inflammation, and colorectal cancer. Intestinal Res 16:338–345. doi:10.5217/ir.2018.16.3.338. PMC6077304 30090032

[14] Bedford A, Gong J 2018 Implications of butyrate and its derivatives for gut health and animal production. Anim Nutr 4:151–159. doi:10.1016/j.aninu.2017.08.010. 30140754 PMC6104520

[15] Bach Knudsen KE, Lærke HN, Hedemann MS, Nielsen TS, Ingerslev AK, Gundelund Nielsen DS, Theil PK, Purup S, Hald S, Schioldan AG, Marco ML, Gregersen S, Hermansen K 2018 Impact of diet-modulated butyrate production on intestinal barrier function and inflammation. Nutrients 10:1499. doi:10.3390/nu10101499. PMC6213552 30322146

[16] Seekatz AM, Theriot CM, Rao K, Chang Y-M, Freeman AE, Kao JY, Young VB 2018 Restoration of short chain fatty acid and bile acid metabolism following fecal microbiota transplantation in patients with recurrent *Clostridium difficile* infection. Anaerobe 53:64. doi:10.1016/j.anaerobe.2018.04.001. 29654837 PMC6185828

[17] Hryckowian AJ, Van Treuren W, Smits SA, Davis NM, Gardner JO, Bouley DM, Sonnenburg JL 2018 Microbiota-accessible carbohydrates suppress *Clostridium difficile* infection in a murine model. Nat Microbiol 3:662–669. doi:10.1038/s41564-018-0150-6. 29686297 PMC6126909

[18] Sworn G 2009 Xanthan gum, p 186–203. *In* Phillips GO, Williams PA (ed), Handbook of hydrocolloids, 2nd ed. Woodhead Publishing, Cambridge, United Kingdom.

[19] Montagne L, Pluske JR, Hampson DJ 2003 A review of interactions between dietary fibre and the intestinal mucosa, and their consequences on digestive health in young non-ruminant animals. Anim Feed Sci Technol 108:95–117. doi:10.1016/S0377-8401(03)00163-9.

[20] Jonathan MC, van den Borne J, van Wiechen P, Souza da Silva C, Schols HA, Gruppen H 2012 *In vitro* fermentation of 12 dietary fibres by faecal inoculum from pigs and humans. Food Chem 133:889–897. doi:10.1016/j.foodchem.2012.01.110.

[21] Reeves AE, Theriot CM, Bergin IL, Huffnagle GB, Schloss PD, Young VB 2011 The interplay between microbiome dynamics and pathogen dynamics in a murine model of *Clostridium difficile* Infection. Gut Microbes 2:145–158. doi:10.4161/gmic.2.3.16333. 21804357 PMC3225775

[22] Chen X, Katchar K, Goldsmith JD, Nanthakumar N, Cheknis A, Gerding DN, Kelly CP 2008 A mouse model of *Clostridium difficile*-associated disease. Gastroenterology 135:1984–1992. doi:10.1053/j.gastro.2008.09.002. 18848941

[23] Collins J, Robinson C, Danhof H, Knetsch CW, van Leeuwen HC, Lawley TD, Auchtung JM, Britton RA 2018 Dietary trehalose enhances virulence of epidemic *Clostridium difficile*. Nature 553:291. doi:10.1038/nature25178. 29310122 PMC5984069

[24] Eyre DW, Didelot X, Buckley AM, Freeman J, Moura IB, Crook DW, Peto TEA, Walker AS, Wilcox MH, Dingle KE 2019 *Clostridium difficile* trehalose metabolism variants are common and not associated with adverse patient outcomes when variably present in the same lineage. EBioMedicine 43:347–355. doi:10.1016/j.ebiom.2019.04.038. 31036529 PMC6558026

[25] Zackular JP, Skaar EP 2018 The role of zinc and nutritional immunity in *Clostridium difficile* infection. Gut Microbes 9:469–476. doi:10.1080/19490976.2018.1448354. 29533126 PMC6219639

[26] Jones RN, Wilson HW, Thornsberry C, Barry AL 1985 *In vitro* antimicrobial activity of cefoperazone-sulbactam combinations against 554 clinical isolates, including a review and β-lactamase studies. Diagn Microbiol Infect Dis 3:489–499. doi:10.1016/s0732-8893(85)80005-5. 2998694

[27] Williams JD 1997 β-Lactamase inhibition and *in vitro* activity of sulbactam and sulbactam/cefoperazone. Clin Infect Dis 24:494–497. doi:10.1093/clinids/24.3.494. 9114205

[28] Seekatz AM, Theriot CM, Molloy CT, Wozniak KL, Bergin IL, Young VB 2015 Fecal microbiota transplantation eliminates *Clostridium difficile* in a murine model of relapsing disease. Infect Immun 83:3838–3846. doi:10.1128/IAI.00459-15. 26169276 PMC4567621

[29] Antonopoulos DA, Huse SM, Morrison HG, Schmidt TM, Sogin ML, Young VB 2009 Reproducible community dynamics of the gastrointestinal microbiota following antibiotic perturbation. Infect Immun 77:2367–2375. doi:10.1128/IAI.01520-08. 19307217 PMC2687343

[30] Theriot CM, Young VB 2015 Interactions between the gastrointestinal microbiome and *Clostridium difficile*. Annu Rev Microbiol 69:445–461. doi:10.1146/annurev-micro-091014-104115. 26488281 PMC4892173

[31] Theriot CM, Bowman AA, Young VB 2016 Antibiotic-induced alterations of the gut microbiota alter secondary bile acid production and allow for *Clostridium difficile* spore germination and outgrowth in the large intestine. mSphere 1:e00045-15. 10.1128/mSphere.00045-15 PMC4863611 27239562

[32] Jarocki P, Targoński Z 2013 Genetic diversity of bile salt hydrolases among human intestinal bifidobacteria. Curr Microbiol 67:286–292. doi:10.1007/s00284-013-0362-1. 23591474 PMC3722454

[33] O’Flaherty S, Briner Crawley A, Theriot CM, Barrangou R 2018 The *Lactobacillus* bile salt hydrolase repertoire reveals niche-specific adaptation. mSphere 3:e00140-18. doi:10.1128/mSphere.00140-18. 29848760 PMC5976879

[34] Kitahara M, Takamine F, Imamura T, Benno Y 2001 *Clostridium hiranonis* sp. nov., a human intestinal bacterium with bile acid 7α-dehydroxylating activity. Int J Syst Evol Microbiol 51:39–44. doi:10.1099/00207713-51-1-39. 11211270

[35] Kang DJ, Ridlon JM, Moore DR 2nd, Barnes S, Hylemon PB 2008 *Clostridium scindens baiCD* and *baiH* genes encode stereo-specific 7α/7β-hydroxy-3-oxo-δ4-cholenoic acid oxidoreductases. Biochim Biophys Acta 1781:16–25. doi:10.1016/j.bbalip.2007.10.008. 18047844 PMC2275164

[36] Thanissery R, Winston JA, Theriot CM 2017 Inhibition of spore germination, growth, and toxin activity of clinically relevant *Clostridium difficile* strains by gut microbiota derived secondary bile acids. Anaerobe 45:86–100. doi:10.1016/j.anaerobe.2017.03.004. 28279860 PMC5466893

[37] Buffie CG, Bucci V, Stein RR, McKenney PT, Ling L, Gobourne A, No D, Liu H, Kinnebrew M, Viale A, Littmann E, van den Brink MR, Jenq RR, Taur Y, Sander C, Cross JR, Toussaint NC, Xavier JB, Pamer EG 2015 Precision microbiome reconstitution restores bile acid mediated resistance to *Clostridium difficile*. Nature 517:205–208. doi:10.1038/nature13828. 25337874 PMC4354891

[38] Weingarden AR, Chen C, Bobr A, Yao D, Lu Y, Nelson VM, Sadowsky MJ, Khoruts A 2014 Microbiota transplantation restores normal fecal bile acid composition in recurrent *Clostridium difficile* infection. Am J Physiol Gastrointest Liver Physiol 306:G310–G319. doi:10.1152/ajpgi.00282.2013. 24284963 PMC3920123

[39] Jenior ML, Leslie JL, Young VB, Schloss PD, Jenior ML, Leslie JL, Young VB, Schloss PD 2017 *Clostridium difficile* colonizes alternative nutrient niches during infection across distinct murine gut microbiomes. mSystems 2:e00063-17. doi:10.1128/mSystems.00063-17. 28761936 PMC5527303

[40] Wilson KH, Perini F 1988 Role of competition for nutrients in suppression of *Clostridium difficile* by the colonic microflora. Infect Immun 56:2610–2614. 3417352 10.1128/iai.56.10.2610-2614.1988 PMC259619

[41] Reeves AE, Koenigsknecht MJ, Bergin IL, Young VB 2012 Suppression of *Clostridium difficile* in the gastrointestinal tracts of germfree mice inoculated with a murine isolate from the family Lachnospiraceae. Infect Immun 80:3786–3794. doi:10.1128/IAI.00647-12. 22890996 PMC3486043

[42] Vincent C, Stephens DA, Loo VG, Edens TJ, Behr MA, Dewar K, Manges AR 2013 Reductions in intestinal *Clostridiales* precede the development of nosocomial *Clostridium difficile* infection. Microbiome 1:18. doi:10.1186/2049-2618-1-18. 24450844 PMC3971611

[43] Moundras C, Behr SR, Rémésy C, Demigné C 1997 Fecal losses of sterols and bile acids induced by feeding rats guar gum are due to greater pool size and liver bile acid secretion. J Nutr 127:1068–1076. doi:10.1093/jn/127.6.1068. 9187619

[44] Levrat-Verny MA, Behr S, Mustad V, Rémésy C, Demigné C 2000 Low levels of viscous hydrocolloids lower plasma cholesterol in rats primarily by impairing cholesterol absorption. J Nutr 130:243–248. doi:10.1093/jn/130.2.243. 10720177

[45] Cox LM, Cho I, Young SA, Anderson WHK, Waters BJ, Hung S-C, Gao Z, Mahana D, Bihan M, Alekseyenko AV, Methé BA, Blaser MJ 2013 The nonfermentable dietary fiber hydroxypropyl methylcellulose modulates intestinal microbiota. FASEB J 27:692–702. doi:10.1096/fj.12-219477. 23154883 PMC3545536

[46] Hoving LR, Katiraei S, Heijink M, Pronk A, van der Wee-Pals L, Streefland T, Giera M, van Dijk KW, van Harmelen V 2018 Dietary mannan oligosaccharides modulate gut microbiota, increase fecal bile acid excretion, and decrease plasma cholesterol and atherosclerosis development. Mol Nutr Food Res 62:e1700942. doi:10.1002/mnfr.201700942. 29665623 PMC6001637

[47] Neyrinck AM, Van Hée VF, Piront N, De Backer F, Toussaint O, Cani PD, Delzenne NM 2012 Wheat-derived arabinoxylan oligosaccharides with prebiotic effect increase satietogenic gut peptides and reduce metabolic endotoxemia in diet-induced obese mice. Nutr Diabetes 2:e28. doi:10.1038/nutd.2011.24. 23154683 PMC3302144

[48] Neyrinck AM, Possemiers S, Verstraete W, De Backer F, Cani PD, Delzenne NM 2012 Dietary modulation of clostridial cluster XIVa gut bacteria (*Roseburia* spp.) by chitin-glucan fiber improves host metabolic alterations induced by high-fat diet in mice. J Nutr Biochem 23:51–59. doi:10.1016/j.jnutbio.2010.10.008. 21411304

[49] Suriano F, Bindels LB, Verspreet J, Courtin CM, Verbeke K, Cani PD, Neyrinck AM, Delzenne NM 2017 Fat binding capacity and modulation of the gut microbiota both determine the effect of wheat bran fractions on adiposity. Sci Rep 7:5621. doi:10.1038/s41598-017-05698-y. 28717237 PMC5514075

[50] Talukdar MM, Michoel A, Rombaut P, Kinget R 1996 Comparative study on xanthan gum and hydroxypropylmethyl cellulose as matrices for controlled-release drug delivery I. Compaction and *in vitro* drug release behaviour. Int J Pharmaceutics 129:233–241. doi:10.1016/0378-5173(95)04355-1.

[51] Dhopeshwarkar V, Zatz JL 1993 Evaluation of xanthan gum in the preparation of sustained release matrix tablets. Drug Dev Industrial Pharm 19:999–1017. doi:10.3109/03639049309062997.

[52] Jackson C, Ofoefule S 2011 Use of xanthan gum and ethylcellulose in formulation of metronidazole for colon delivery. J Chem Pharm Res 3:11–20.

[53] Andreopoulos AG, Tarantili PA 2001 Xanthan gum as a carrier for controlled release of drugs. J Biomater Appl 16:34–46. doi:10.1106/XBFG-FYFX-9TW9-M83U. 11475358

[54] Theriot CM, Koumpouras CC, Carlson PE, Bergin II, Aronoff DM, Young VB 2011 Cefoperazone-treated mice as an experimental platform to assess differential virulence of *Clostridium difficile* strains. Gut Microbes 2:326–334. doi:10.4161/gmic.19142. 22198617 PMC3337121

[55] Leslie JL, Vendrov KC, Jenior ML, Young VB 2019 The gut microbiota is associated with clearance of *Clostridium difficile* infection independent of adaptive immunity mSphere 4:e00698-18. 30700514 10.1128/mSphereDirect.00698-18 PMC6354811

[56] Nadkarni MA, Martin FE, Jacques NA, Hunter N 2002 Determination of bacterial load by real-time PCR using a broad-range (universal) probe and primers set. Microbiology 148:257–266. doi:10.1099/00221287-148-1-257. 11782518

[57] Kozich JJ, Westcott SL, Baxter NT, Highlander SK, Schloss PD 2013 Development of a dual-index sequencing strategy and curation pipeline for analyzing amplicon sequence data on the MiSeq Illumina sequencing platform. Appl Environ Microbiol 79:5112. doi:10.1128/AEM.01043-13. 23793624 PMC3753973

[58] Segata N, Izard J, Waldron L, Gevers D, Miropolsky L, Garrett WS, Huttenhower C 2011 Metagenomic biomarker discovery and explanation. Genome Biol 12:R60. doi:10.1186/gb-2011-12-6-r60. 21702898 PMC3218848

